# Effects of a hot ambient operating theatre on manual dexterity, psychological and physiological parameters in staff during a simulated burn surgery

**DOI:** 10.1371/journal.pone.0222923

**Published:** 2019-10-16

**Authors:** Zehra Palejwala, Karen Wallman, MK Ward, Cheryl Yam, Tessa Maroni, Sharon Parker, Fiona Wood

**Affiliations:** 1 School of Human Sciences (Sports Science, Exercise and Health), The University of Western Australia, Perth, Western Australia, Australia; 2 Centre for Transformative Work Design, Faculty of Business and Law, Curtin University, Western Australia, Australia; 3 Fiona Stanley Hospital, Perth, Western Australia, Australia; Medical University Graz, AUSTRIA

## Abstract

**Objectives:**

Hot environmental conditions can result in a high core-temperature and dehydration which can impair physical and cognitive performance. This study aimed to assess the effects of a hot operating theatre on various performance, physiological and psychological parameters in staff during a simulated burn surgery.

**Methods:**

Due to varying activity levels, surgery staff were allocated to either an Active (n = 9) or Less-Active (n = 8) subgroup, with both subgroups performing two simulated burn surgery trials (CONTROL: ambient conditions; 23±0.2°C, 35.8±1.2% RH and HOT: 34±0°C, 28.3±1.9% RH; 150 min duration for each trial), using a crossover design with four weeks between trials. Manual dexterity, core-temperature, heart-rate, sweat-loss, thermal sensation and alertness were assessed at various time points during surgery.

**Results:**

Pre-trials, 13/17 participants were mildly-significantly dehydrated (HOT) while 12/17 participants were mildly-significantly dehydrated (CONTROL). There were no significant differences in manual dexterity scores between trials, however there was a tendency for scores to be lower/impaired during HOT (both subgroups) compared to CONTROL, at various time-points (Cohen’s *d* = -0.74 to -0.50). Furthermore, alertness scores tended to be higher/better in HOT (Active subgroup only) for most time-points (p = 0.06) compared to CONTROL, while core-temperature and heart-rate were higher in HOT either overall (Active; p<0.05) or at numerous time points (Less-Active; p<0.05). Finally, sweat-loss and thermal sensation were greater/higher in HOT for both subgroups (p<0.05).

**Conclusions:**

A hot operating theatre resulted in significantly higher core-temperature, heart-rate, thermal sensation and sweat-loss in staff. There was also a tendency for slight impairment in manual dexterity, while alertness improved. A longer, real-life surgery is likely to further increase physiological variables assessed here and in turn affect optimal performance/outcomes.

## Introduction

Over 1.1 million people worldwide each year are affected by burns, with 70% of hospitalisations requiring surgery [1]. During a burn surgery, it is common practice to maintain the ambient temperature in the operating room (OR) between 30–40°C [2] so to prevent patients experiencing inadvertent intraoperative hypothermia (core temperature: Tc <36°C [3]), a phenomenon that occurs frequently in elective surgeries [4]. The negative effects of inadvertent hypothermia during surgery affect almost all aspects of the post-operative course from patient recovery time to cardiac morbidity [5]. Consequently, maintaining the OR at a high ambient temperature during burn surgeries enhances the likelihood of better outcomes for the patient [6].

An issue related to staff working in a hot surgical environment is that heat exposure (combined with physical activity) can raise an individual’s Tc above the normal resting level of ~37.0°C [7]. While a slight increase in Tc (to ~37.5°C), as a result of a warm-up, has been found to improve subsequent physical/exercise performance [8], hyperthermia can occur at a Tc of ~38°C, which can result in heat strain [9] and impaired subsequent exercise performance [10]. Further, a Tc >39.4°C is associated with heat illnesses [11], impaired central nervous system motor drive, reduced force output [12] and premature fatigue during exercise [13]. Moreover, an increase in Tc during hot environmental conditions leads to an increase in sweat loss, which can result in dehydration [14]. Notably, dehydration of ≥2% of body-mass has been reported to impair: exercise performance [15]; concentration; routine mental work capacity; arithmetic ability; and short-term memory [16–17]; compared to a hydrated state. These are important considerations for surgical staff regularly faced with complex scenarios when working in an OR. Furthermore, clothing worn by staff during burn surgeries typically consists of multiple layers, with minimal skin exposed. This combined with minimal air flow, due to fans not being used in a burns OR due to their association with a greater risk of surgical site infection [18], can result in an increase in Tc and greater sweat loss [19].

Of relevance are the effects of working in a hot OR on physical and psychological parameters in staff. Specifically, manual dexterity plays a critical role in a surgical environment due to the important role of the hands when performing surgical manoeuvres and in the manipulation of surgical equipment [20]. To date, no studies have assessed manual dexterity performance (Purdue pegboard test) in heat compared to a normothermic environment, suggesting that further studies are needed here. Furthermore, a high level of alertness is of extreme importance in surgical staff where physical and cognitive performance play important roles in relation to patient outcomes. Importantly, while subjective alertness scores (visual analog scale) have been reported to improve over the course of a day due to a natural increase in Tc that occurs as a result of circadian rhythm (37.8 vs 36.8°C: [21]), other studies have reported impaired alertness associated with hot ambient conditions whilst driving [22] and exercising [23], with Tc values in these studies not assessed/reported.

To date, only two studies have investigated the effects of high ambient OR temperatures on surgical staff. Hakim et al. [24] examined the effects of varying OR temperatures (21°C, 23°C, 26°C: 30 min per trial) on clinical and cognitive performance in OR personnel (6 men, 16 women) using a psychomotor vigilance test device (assesses response speed to a visual stimulus) and self-report questionnaires, with the duration between trials not reported. These researchers found no difference in reaction time scores between the three trials but found that self-reported performance scores and physical demand and frustration were significantly higher in the warmer OR (26°C) compared to the other two trials. Further, Berg et al. [25] assessed the effects of high ambient OR temperature (26°C) compared to 19°C on surgical performance (peg-transfer and knot tying tasks) and cognitive stress (Surgical Task Load Index) in OR staff (21 males) during a simulated laparoscopic surgical task (30 min duration). No significant differences in surgical performance were reported between the two trials, however in the warmer trial, levels of physical demand and distractions were reported to be greater [25]. Importantly, burn surgeries typically last longer than 2 hours [26] and are conducted at temperatures between 30–40°C [2], suggesting that the findings by Hakim et al. [24] and Berg et al. [25] are not true reflections of what occurs in a typical burn surgery. Further research is needed to determine the effect of a hot environment (30–40°C) over a longer surgery time (>30 mins) on OR staff working during a burn surgery.

Therefore, the aim of this study was to assess the effects of a high ambient OR on manual dexterity, alertness, thermal sensation and physiological parameters in staff during a simulated burn surgery (150 mins duration), compared to normothermic ambient conditions. As surgery staff include those who stand and move for the entire surgery, compared to some staff who generally remain seated, it was decided to allocate staff to either an Active or Less Active subgroup as higher activity levels can increase Tc and heart-rate compared to lower levels ([27] see Methods for more detail). It was hypothesised that a surgery performed in a hot OR would result in: improved alertness; higher thermal sensation, Tc and heart-rate; greater sweat loss; and impaired manual dexterity in the Active subgroup, compared to surgery performed in a normothermic environment.

## Materials and methods

### Participants and overview of the study

**Fig 1:**
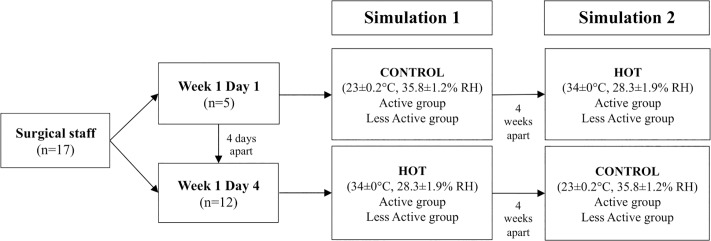
Overview of study design.

Surgical and hospital staff (n = 17, [male = 8, female = 9], staff characteristics provided in detail later), consisting of surgeons (n = 4), scrub nurses (n = 6), anaesthetists (n = 3), anaesthetist technicians (n = 1), physiotherapists (n = 2) and an occupational therapist (n = 1) were recruited from Fiona Stanley Hospital, Western Australia, for participation in this study. Participants volunteered to attend either the initial surgery simulation (CONTROL; 23±0.2°C, relative humidity [RH] 35.8±1.2%) held on day 1/week 1 (n = 5, males = 3, females = 2) or the second trial (HOT; 34.0°C, 28.3±1.9% RH) held on day 4/week 1 (n = 12, males = 5, females = 7) based on their work availability. Participants worked in the opposite ambient conditions (crossover design) exactly four weeks later (an attempt to minimize any confounding effects of the menstrual cycle) on the same day (i.e., day 1/week 4 and day 4/week 4) and at the same time as the first trials. Furthermore, as higher levels of physical activity result in a higher metabolism and an associated increase in Tc and heart-rate compared to resting values [27], it was decided to allocate staff to one of two subgroups based on each staff member’s expected activity levels. Staff who anticipated to be standing for the majority of surgery time (approx ≥75%) performing active tasks were allocated to the Active subgroup (four surgeons, four scrub nurses and an anaesthetist technician; average age 36.0±9.1 y, height 167.0±6.3 cm, body-mass 68.8±14.4 kg), while those who were expected to be seated for the majority of surgery time (approx ≥75%), performing minimal activity were allocated to the Less-Active subgroup (two physiotherapists, three anaesthetists, two scrub nurses and an occupational therapist; average age 35.1±4.3 y, height 175.3±8.9 cm, body-mass 83.7±18.0 kg). This allocation was agreed upon by three senior medical staff (>20 years experience in burn surgeries), with standing time and activity levels of participants later confirmed by researcher observations during the simulations. Fig 1 provides an overview of the study.

Participants were recruited during June, 2018, while the study was conducted from July to August, 2018 (winter months) in Perth, Western Australia where average temperatures ranged between 8.7°C to 18.9°C [28]. Therefore participants were unlikely to be heat acclimatised, an effect known to improve physical performance in hot environments. In addition, questionnaires regarding previous heat exposure (i.e., surgical environments/recent holidays in hot climates) were answered so to check for any prior acclimatisation/acclimation (none were found). Participants wore the same clothing for each simulation (scrubs, surgical gowns, enclosed shoes, gloves, hair nets, aprons and face shields if required) and replicated their food and fluid intake, as well as physical activities undertaken for the 24 h period prior to each simulation (based on 24 h food and activity diaries kept by each participant). Prior to starting the trials, all participants were provided with the details of the study and gave their informed consent, with ethical approval granted by the Human Research Ethics Committee of the University of Western Australia (RA/4/20/4520) and the South Metropolitan Health Service Human Research Ethics Committee (PRN RGS0000000833).

### Familiarisation session

Participants attended a familiarisation session approximately one week prior to their first simulated surgery. Anthropometric measurements including height (cm) and body-mass (kg) were assessed and participants were given a food and activity diary to complete for the 24 h period prior to the first surgery simulation. Participants were also provided with a Tc capsule (CorTemp, HQ Inc., Palmetto, USA) to ingest eight hours prior to the simulation (as per manufacturer’s instructions). Participants then completed the Purdue Pegboard test (used to assess manual dexterity). Further, as T_c_ is affected by menstrual cycle phase [29], female participants provided information on their menstrual cycle patterns (i.e. first day of last menstrual period and length of menstrual cycle) and the type of contraception they were using at the time of the study (i.e. pill, intra-uterine device [IUD] or none) in order to determine which menstrual cycle phase they were in during each simulation.

### Surgical simulation protocol and variables assessed

Upon arrival to each simulation, a urine sample was collected (mid-stream in a sterile container approximately one hour prior) to determine individual urinary specific gravity (USG) for hydration status assessment. Indices for hydration status were taken from a study by Kavouras [30] where USG scores <1.010 represented individuals who were ‘well hydrated’, scores between 1.010–1.020 represented ‘minimal hydration’, scores between 1.021–1.030 represented ‘significant dehydration’, while scores >1.030 represented ‘serious hydration’. Next, nude body-mass was measured to the nearest 0.1 kg using a digital platform scale (SOEHNLE, Style sense comfort 100, Germany). Participants were then fitted with a heart-rate monitor (Polar RS400, Finland) and entered the OR where all baseline measures (Tc, heart-rate, manual dexterity, thermal sensation and alertness) were assessed at varying intervals (details provided later).

While the surgical simulation ended after 2.5 h, the actual surgical task was designed to last for ~3 h. This earlier termination time ensured that all staff were actively involved in their given tasks for the duration of the simulation. The surgical simulation was designed to replicate a surgery of a patient with burns to 20% of their total body surface area (burns to the face, arms, legs and the abdomen; first simulation day 1/ week 1) or 40% of total body surface area (face and arms but a greater proportion of burns to the legs and abdomen; second simulation day 4/ week 1). The difference in burn surface areas between days was designed to cater for the larger surgical team (n = 12) participating during the second simulation compared to the smaller team involved with the first simulation (n = 5). The simulations performed four weeks later were of the same design to the first simulations in respect to burn surface areas and location of burns, but with some minor adaptations, meaning that no two surgeries were exactly the same but required similar surgical skills, effort and attention. An anatomically correct mannequin typically used as the ‘patient’ in medical training was used during the simulations and was surgically dressed previously by an experienced burn surgeon (>20 y experience in burn surgeries) with different coloured bindings representing different degrees of injury, with each layer related to the layers of the skin removed serially to mimic a real life surgical procedure. During the simulations, the participants fulfilled their usual surgical and hospital roles and responsibilities within the OR in order to create a real-life scenario. Once all the initial baseline measurements were made, no fluid or food were ingested, nor were any toilet breaks taken until after the final measurements were recorded at the end of each surgery simulation.

The Purdue Pegboard test (Model 32020, J.A Preston Corporation, New York; [31]) was used to assess fine manual dexterity of the hands and involved placing as many pins (one at a time) into a row of holes in 30 s, first using the dominant hand (30 s) and then the non-dominant hand (30 s) with a break in-between hands of ~5 s. Test-retest reliability assessment of the Purdue Pegboard task performed prior to the study (n = 10) resulted in a typical error (TE) score of ±0.5, with a coefficient of variation (CV) of 3.1% for the dominant hand scores and a TE of ±0.7 (CV: 4.4%) for the non-dominant hand.

Thermal sensation was rated using an 8-point scale (0 = unbearably cold to 8 = unbearably hot; [32] while alertness was rated using the 7-point Stanford sleepiness scale (1 = no longer fighting sleep, sleep onset soon, having dream like thoughts to 7 = wide awake, vital, alert or feeling active; [33]). All measures were assessed at the start and end of the surgery simulation (0 and 150 min marks). Further, Tc and heart-rate were measured every 15 min during the simulation, while manual dexterity and thermal sensation were assessed at every 30 min interval of the simulation. On completion of the simulation, participants exited the OR, removed all clothing, heart-rate monitors and watches, towel dried and nude body-mass was re-measured to determine sweat loss which was calculated as: pre nude body mass–post nude body mass.

### Statistical analysis

All statistical analysis was conducted using IBM SPSS statistics version 20.0. Mixed /split plot two way ANOVAs were used to analyse differences between subgroups (Active and Less Active). Further, two-way repeated-measures ANOVAs were used to analyse differences between physiological variables (Tc and heart-rate), as well as the performance variables (manual dexterity, thermal sensation and alertness) between Active (HOT) versus Active (CONTROL) and Less-Active (HOT) versus Less-Active (CONTROL), across the four simulations at each time point as described in the Methods section. Paired samples t-tests assessed USG and sweat loss. Where main or interaction effects occurred, follow-up post hoc comparisons using Bonferroni adjustments and paired sample t-tests were used. Statistical significance was accepted at p≤ 0.05 for all variables. In addition, inferential statistical analysis was performed using Cohen’s *d* effect sizes (ES; [34]) with only moderate and large (≥0.5) ES reported. Mean difference ± 95% confidence intervals (CI) were also calculated to assess the magnitude of these differences. All values are expressed as mean ± SD.

## Results

Environmental conditions were 34±0°C, 28.3±1.9% RH for the HOT trials and 23±0.2°C, 35.8±1.2% RH for the CONTROL trials. There was no significant order effect for manual dexterity performance (p>0.05) over the two trials. Of the nine female participants, one reported being on the contraceptive pill (28 day Chelsea-35 ED, monophasic), one participant was post-menopausal, three used an IUD (natural menstrual cycle is generally unaffected by the presence of an IUD and remains ~28 days in length; [35]), while four participants were not using any contraception. Of these four, only one was calculated to have a differing menstrual cycle phase for the second simulation (luteal) compared to the first simulation (menstrual phase). Of relevance, there were no significant differences or moderate to large ES between trials (HOT and CONTROL) for either subgroup (Active and Less Active) for baseline T_c_ values.

### Active and less-active subgroup results and synopsis

Prior to the main analyses (i.e. HOT [Active] versus CONTROL [Active] and HOT [Less-Active] versus CONTROL [Less-Active], Tc and heart-rate values were compared between staff allocated to the Active and Less-Active subgroups.

In respect to Tc, there were no interaction effects (p>0.05) for either trial. However, in the CONTROL trial, Tc in the Active subgroup increased by 0.29±0.45°C from baseline to peak Tc (135 min mark, *d* = 0.88 [-0.13, 1.80), yet decreased in the Less-Active subgroup by 0.04±0.30°C from baseline to peak Tc (135 min mark). Further, Tc was higher in the Active compared to the Less-Active subgroup at 120, 135 and 150 min (*d* = 0.60 [-0.4, 1.54] to 0.77 [-0.25, 1.72]). During the HOT trial, Tc increased over the course of the simulation by 0.37±0.32°C in the Active subgroup and by 0.33±0.26°C in the Less-Active subgroup, with Tc being higher in the Active subgroup for every 15 min interval recorded from 30 min to 150 min (*d* = 0.55 [-0.45, 1.49] to 1.02 [-0.04, 1.97]). In respect to heart-rate, there was no interaction effect between subgroups (p>0.05) for the CONTROL trial. However, heart-rate in the CONTROL trial decreased over the course of the simulation (0–150 min) by 2±11 bpm in the Active subgroup and by 5±7 bpm in the Less-Active subgroup with a moderate ES (*d* = 0.55 [-0.45, 1.49) found at the 45 min mark (higher heart-rate in the Active subgroup). During the HOT trial, there was an interaction effect between subgroups (p<0.05). In addition, heart-rate increased over the course of the simulation by 14±10 bpm in the Active subgroup (*d* = 1.27 [0.20, 2.21]) and by 4±9 bpm in the Less-Active subgroup, with heart-rate being higher in the Active subgroup at 75, 90, 105 and 150 min (*d* = 0.55 [-0.44, 1.49] to 0.8 [-0.23, 1.74).

As noted in the Methods section, the above analysis was performed due to the fact that some staff roles/duties resulted in higher levels of activity than others (i.e., surgeons who stood and were active throughout surgery compared to anaesthetists who were mostly seated), and that this was expected to result in higher Tc and heart-rate values. Results found here support the use of employing Active and Less-Active subgroups in this study. This approach should be considered in respect to future studies that assess the impact of ambient temperature on physical performance in staff.

### Manual dexterity performance

There were no significant interaction effects (p>0.05) between trials for either subgroup for manual dexterity performance for either the dominant or non-dominant hand. In respect to the dominant hand, scores for the pegboard test improved over the course of the simulation in the Active subgroup during both the HOT and CONTROL trials (*d* = 0.85 [-0.15, 1.77] and *d* = 0.53 [-0.44, 1.44], respectively), and in the HOT trial only for the Less-Active subgroup (*d* = 0.71 [-0.33, 1.68]; Table 1). Interestingly, scores for the Active subgroup were lower during the HOT compared to the CONTROL trial at the 30 min mark (*d* = -0.54 [-1.45, 0.43]). In respect to the non-dominant hand, scores also improved over time in both subgroups during both the HOT and CONTROL trials (*d* = 0.52 [-0.51, 1.48] to 1.27 [0.21, 2.22]; Table 1). However, during the simulation, scores for the non-dominant hand were lower in the HOT compared to the CONTROL trial for the Active subgroup at 90 min (*d* = -0.54 [-1.45, 0.42]) and at the 30 and 90 min mark (*d* = -0.74 [-1.71, 0.31] and *d* = -0.5 [-1.47, 0.52] respectively) for the Less-Active subgroup. Conversely, scores for the Less-Active subgroup were higher in the HOT compared to the CONTROL trial at the 60 min mark (*d* = 0.58 [-0.45, 1.55]).

**Table 1:** Mean ± SD Purdue pegboard scores for the dominant and non-dominant hand at each 30-minute interval during the simulation for the Active (n = 9) and the Less-Active (n = 8) subgroups in the CONTROL and HOT trials. Unit = number of pins inserted in holes on the pegboard in 30 s.

DOMINANT HAND						
	0 min	30 min	60 min	90 min	120 min	150 min
**Active group**^a^						
CONTROL (pins)	17 ± 2	17 ± 2	17 ± 2	18 ± 2	17 ± 2	18 ± 2^b^
HOT (pins)	16 ± 2	16 ± 2^c^	17 ± 2	18 ± 2	18 ± 2	18 ± 2^b^
**Less-Active group**						
CONTROL (pins)	16 ± 2	17 ± 1	17 ± 1	17 ± 1	17 ± 1	17 ± 2
HOT (pins)	16 ± 1	17 ± 1	17 ± 1	17 ± 2	17 ± 1	17 ± 1^b^
**NON-DOMINANT HAND**						
**Active group**^a^						
CONTROL (pins)	15 ± 2	16 ± 2	17 ± 2	17 ± 2	17 ± 1	17 ± 2^b^
HOT (pins)	15 ± 2	16 ± 2	17 ± 2	16 ± 2^c^	17 ± 2	17 ± 1^b^
**Less Active group**						
CONTROL (pins)	15 ± 2	15 ± 1	15 ± 1	16 ± 2	15 ± 2	16 ± 2^b^
HOT (pins)	15 ± 1	14 ± 1^c^	16 ± 2^c^	15 ± 2^c^	15 ± 1	16 ± 1^b^

^a^ indicates significant main effect for time (p<0.05);

^b^ indicates moderate to large effect sizes between time points 0 min and 150 min (*d* = 0.52 to 1.27);

^c^ indicates moderate effect sizes between HOT and CONTROL trials (*d* = -0.74 to 0.58).

### Psychological variables

While no significant interaction effect (p>0.05) was found for thermal sensation for the Active subgroup during the HOT compared to the CONTROL trials, moderate to large ES demonstrated that thermal sensation increased in this subgroup over the course of the simulation during both the HOT (*d* = 0.63 [-0.35, 1.54]) and CONTROL trials (*d* = 1.69 [0.55, 2.68]). Further, in respect to the Active subgroup, there was a significant main effect for trial (p<0.05) with participants reporting higher scores, ranging from 5.5 (warm-hot) to 6.0 (hot), during the HOT trial compared to lower scores; 3.0 (cool) to 4.5 (comfortable-warm) in the CONTROL trial, with this supported by large ES for every time point assessed (*d* = 1.56 [0.44, 2.53] to 3.54 [1.93, 4.80]). No significant interaction effect was found for thermal sensation in the Less-Active subgroup (p>0.05) between trials. However, there was a significant main effect for trial (p<0.05), with scores being higher overall for the HOT trial, ranging from 6.0 (hot) to 6.5 (hot-very hot) over time compared to scores of 4.5 (comfortable-warm) for every time point assessed, apart for the 60 min mark (5.0 = warm) in the CONTROL trial. The higher scores in the HOT compared to the CONTROL trial (Less-Active subgroup) were also supported by large ES for every time point assessed (*d* = 1.47 [0.30, 2.48] to 2.48 [1.07, 3.62]). Further, thermal sensation increased over time in the Less-Active subgroup during the HOT trial only (*d* = 0.55 [-0.47, 1.52]).

While there was no significant interaction effect between trials (p>0.05) for alertness scores, these scores increased over time in the Active subgroup during the CONTROL trial only, with this supported by a large ES (*d* = 0.83 [-0.17, 1.75]). Notably, scores for this subgroup were higher overall in the HOT compared to the CONTROL trial (main effect for trial approaching significance; p = 0.06), with moderate to large ES found between trials at baseline, 60, 90 and 120 min (*d* = 0.83 [-0.17, 1.75] to 1.81 [0.64, 2.81]). Specifically, scores ranged from 5 (responsive but not fully alert, awake, but relaxed) to 6 (able to concentrate, functioning at high levels, but not at peak) in the CONTROL trial compared to 6 (defined above) to 7 (wide awake, vital, alert, or feeling active) in the HOT trial. In respect to the Less-Active subgroup, there was no significant interaction effect (p>0.05), however scores for alertness increased over time in the CONTROL trial only (*d* = 0.95 [-0.13, 1.93]). Scores ranged from 5 to 6 in both trials and were the same for both trials at each time point, apart for the 150 min mark where scores were lower during the HOT trial (i.e. 5) compared to the CONROL trial (i.e. 6) (*d* = -1.17 [-2.16, -0.05]).

### Physiological variables

While there was no significant interaction effect (p>0.05) for Tc in the Active subgroup, Tc increased over time during the HOT and CONTROL trials (main effect for time: p<0.05; and *d* = 1.33 [0.26, 2.28] and *d* = 0.72 [-0.27, 1.64] respectively; Table 2). Notably, there was also a significant main effect for trial in the Active subgroup (p<0.05), with Tc being overall higher in the HOT compared to the CONTROL trial, with this also supported by moderate to large ES found at all time points from the 15–150 min mark (*d* = 0.57 [-0.40, 1.49] to 1.27 [0.20, 2.21]). In respect to the Less-Active subgroup, Tc increased over time (main effect for time: p<0.05), with a large ES found between baseline and the 150 min mark in the HOT trial only (*d* = 1.10 [0.00, 2.08]; Table 2). Further, there was a significant interaction effect (p<0.05) in this subgroup, with Tc being significantly higher in the HOT compared to the CONTROL trial from 90–150 min marks (p<0.05; *d* = 0.7 [-0.34, 1.67] to 1.07 [-0.03, 2.05] for 90 to 150 min).

**Table 2:** Mean±SD core temperature values between the CONTROL and HOT trials at each 15-minute interval during the simulation for the Active subgroup (n = 9) and the Less Active subgroup (n = 8).

Core Temperature (°C)											
	0 min	15 min	30 min	45 min	60 min	75 min	90 min	105 min	120 min	135 min	150 min
**ACTIVE SUBGROUP**	37.22±0.40	37.25±0.37	37.33±0.34	37.34±0.33	37.39±0.31	37.45±0.29	37.42±0.31	37.50±0.23	37.48±0.26	37.51±0.24	37.46±0.25
CONTROL^e^											
HOT^a^^,^^b^^,^^e^	37.39±0.31	37.42±0.20^f^	37.58±0.23^f^	37.70±0.25^f^	37.75±0.21^f^	37.75±0.21^f^	37.80±0.23^f^	37.77±0.27^f^	37.78±0.24^f^	37.75±0.25^f^	37.76±0.24^f^
**LESS ACTIVE SUBGROUP**											
CONTROL	37.29±0.35	37.29±0.44	37.29±0.46	37.29±0.51	37.36±0.52	37.38±0.44	37.38±0.43	37.33±0.41	37.26±0.43	37.25±0.37	37.25±0.32
HOT^a^^,^^c^^,^^e^	37.28±0.38	37.28±0.41	37.34±0.36	37.41±0.32	37.50±0.28	37.53±0.29	37.58±0.31^d^	37.60±0.32^d^^,^^f^	37.59±0.30^d^^,^^f^	37.57±0.29^d^^,^^f^	37.61±0.31^d^^,^^f^

^a^ indicates a significant main effect for time (P<0.05);

^b^ indicates a significant main effect for trial (p<0.05);

^c^ indicates a significant interaction effect (p<0.05);

^d^ indicates time points at which significant differences between trials were found (p<0.05);

^e^ indicates moderate to large effect size between time points 0 min and 150 min (*d* = 0.72 to 1.33);

^f^ indicates moderate to large effect size between HOT and CONTROL trials (*d* = 0.57 to 1.27).

In respect to heart-rate, there was a significant main effect for time and trial and a significant interaction effect (p<0.05, Table 3) between the HOT and CONTROL trials for the Active subgroup. Results showed that HR increased over time in the HOT trial only (*d* = 1.27 [0.20, 2.21]), with HR being significantly higher (p<0.05) in the HOT compared to the CONTROL trial at each time point from the 60 min to 150 min mark, with this also supported by large ES for all time points between 45 and 150 min (*d* = 0.88 [-0.13, 1.80] to 1.38 [0.30, 2.34]). In respect to the Less-Active subgroup, there was a significant main effect for time and a significant interaction effect (p<0.05), with heart-rate being significantly higher in the HOT compared to the CONTROL trial at the 60, 120 and 135 min marks (p<0.05), with moderate to large ES also found at the 60 min mark and from the 105–150 min mark (*d* = 0.51 [-0.52, 1.47] to 0.96 [-0.12, 1.94]; Table 3).

**Table 3:** Mean±SD heart-rate values between the CONTROL and HOT trials at each 15-minute interval during the simulation for the Active subgroup (n = 9) and the Less Active subgroup (n = 8).

Heart Rate (bpm)											
	0 min	15 min	30 min	45 min	60 min	75 min	90 min	105 min	120 min	135 min	150 min
**ACTIVE** **SUBGROUP**											
CONTROL	81±13	83±13	93±19	88±7	83±11	83±12	84±14	78±13	79±10	79±9	79±11
HOT^a^^,^^b^^,^^c^^,^^e^	80±9	84±12	97±20	96±11^f^	97±15^d^^,^^f^	98±16^d^^,^^f^	97±16^d^^,^^f^	95±15^d^^,^^f^	96±17^d^^,^^f^	94±12^d^^,^^f^	94±13^d^^,^^f^
**LESS ACTIVE** **SUBGROUP**											
CONTROL	83±20	85±20	89±19	80±20	77±17	78±16	77±17	76±16	74±13	74±12	78±16
HOT^a^^,^^c^	82±17	84±21	92±21	90±22	89±18^d^^,^^f^	86±19	83±19	85±18^f^	88±16^d^^,^^f^	87±20^d^^,^^f^	86±16^f^

^a^ indicates a significant main effect for time (P<0.05);

^b^ indicates a significant main effect for trial (p<0.05);

^c^ indicates a significant interaction effect (p<0.05);

^d^ indicates time points at which significant differences between trials were found (p<0.05);

^e^ indicates moderate to large effect size between time points 0 min and 150 min (*d* = 1.27);

^f^ indicates moderate to large effect size between HOT and CONTROL trials (*d* = 0.51 to 1.38).

Hydration status was also measured prior to the start of each simulated surgery. Results for USG did not differ between the HOT and CONTROL trials for either subgroup (average score for HOT-Active = 1.010±0.005, CONTROL-Active = 1.015±0.007, HOT-Less-Active = 1.015±0.007 and CONTROL-Less-Active = 1.010±0.007; p>0.05). Notably, USG results for the Active subgroup prior to the CONTROL trial demonstrated that two individuals were well hydrated; six were mildly dehydrated, while one was significantly dehydrated [30]. Prior to the HOT trial (Active subgroup), two participants were well hydrated and seven were mildly dehydrated [30]. Furthermore, prior to the CONTROL trial for the Less-Active subgroup, three participants were well hydrated and five were mildly dehydrated, whereas prior to the HOT trial, two were well hydrated, five were mildly dehydrated while one was moderately dehydrated [30].

Finally, pre and post-trial nude body-mass indicated that sweat loss was significantly greater during the HOT trials (Active = 0.6±0.3 kg, ~0.9% loss overall, Less-Active = 0.8±0.4 kg, ~1.0% loss overall) compared to the CONTROL trials (Active = 0.2±0.2 kg, ~0.3% loss overall, Less-Active = 0.2±0.2 kg, ~0.2% loss overall) for both subgroups (p< 0.05).

## Discussion

This study aimed to assess the effects of a hot ambient OR on manual dexterity, psychological and physiological parameters in surgical staff (allocated to either an Active or Less-Active subgroup) during a simulated burn surgery, compared to normothermic ambient conditions. While not statistically significant, results for manual dexterity performance were found to be slightly impaired in the HOT compared to the CONTROL trial at various time points in both subgroups, as supported by Cohen’s *d* ES. Additionally, thermal sensation scores were overall significantly higher in the HOT compared to the CONTROL trial for both subgroups, while alertness scores were higher in the HOT compared to the CONTROL trial for the Active group only (moderate to large ES). Core temperature and heart-rate values were also significantly higher in HOT compared to CONTROL at various time points or overall in both subgroups, while sweat loss was significantly greater in the HOT trial for both subgroups compared to CONTROL. These results provide some support for our hypothesis.

Manual dexterity improved over the course of the simulation in both hands and in both subgroups and trials with the exception of the CONTROL trial (Less-Active subgroup, dominant hand). This overall improvement may have been related to the beneficial effects of a passive/active warm-up (due to exposure to heat and/or physical activity) as reflected by Tc values that slowly increased over the course of the simulation (ranging from: 37.22–37.76°C) in all trials except for the CONTROL trial (Less Active subgroup), where Tc values actually decreased over time. Temperature related benefits of a passive or active warm-up on subsequent exercise have been attributed to various mechanisms such as decreased stiffness of muscle fibres during contraction [36] reduced muscle and joint viscous resistance [37], greater release of oxygen from haemoglobin and myoglobin [38], the speeding of oxygen kinetics [39], and increased neural conduction rates [40].

Results in the current study are similar to that by Maroni et al. [41], who reported improved pegboard performance over the course of a 30 min exercise trial performed in the heat (35±1.2°C, 52.5±7.4% RH); however no comparisons were made by these researchers to pegboard performance undertaken in normothermic conditions. Notably, Noguchi et al. [42] reported improved manual dexterity performance using the Purdue Pegboard test over the course of five consecutive trials as being due to a practice effect. This explanation is unlikely to apply to this current study due to performance not improving in the CONTROL trial (Less-Active subgroup), unless a practice effect only occurs in conjunction with an increase in Tc (as seen in all other trials/subgroups where Tc increased and performance improved).

Results from this study also indicated a tendency for impaired manual dexterity performance in the HOT compared to the CONTROL trial in both subgroups at a number of time-points during the simulation. This result is unlikely due to the significantly higher Tc values recorded during the HOT compared to CONTROL trials for both subgroups, as these values did not reach hyperthermic levels (≥38°C), which has been reported to impair exercise performance [10]. Participants did however feel hotter (significantly higher thermal sensation scores) in the HOT compared to the CONTROL trials, with thermal discomfort previously found to have an adverse effect on the efficiency and quality of work [43].

Another explanation for results found in the current study may relate to the 13/17 participants (76%) found to be mildly-moderately dehydrated prior to commencing the HOT trials, with subsequent sweat loss over the course of the simulation being significantly higher in the HOT compared to the CONTROL trials. Higher sweat loss in participants in the HOT trial was most likely due to the combination of the hot ambient conditions and the wearing of clothing that restricted cooling. Importantly, Below et al. [44] reported that a 2% loss in body-mass due to sweat loss in previously hydrated participants resulted in a 6.5% impairment in subsequent exercise (cycling) performance. Of relevance, participants in the HOT trials here lost ~0.9% body-mass (Active) and ~1.0% body-mass (Less-Active) during the simulation, with total body-mass loss from each participants’ original hydrated state being incalculable due to most participants commencing the trials in an already dehydrated state. A deteriorating hydration status found here, combined with significantly higher thermal sensation values (likely due to sweat loss) recorded during the HOT surgery simulations may together have played a role in the slight impairment found for manual dexterity performance observed during these trials compared to CONTROL.

Nonetheless, the question arises of why manual dexterity was only impaired at some but not all time points during the simulation. A possible explanation for this may relate to the work task performed immediately prior to each individual pegboard test. Specifically, if the work task was more taxing/stressful than previous tasks, then this either alone or combined with a significantly higher thermal sensation and dehydration may have negatively impacted subsequent manual dexterity performance. Some support for this premise is provided by Van Cutsem et al. [45], who reported that performing a reaction time test in the heat (30°C, 30% RH), as opposed to watching a documentary (a less stressful task) in the same ambient conditions, resulted in higher fatigue scores. Importantly, fatigue has been found to decrease task motivation, result in longer reaction time responses and poor judgement, impair concentration and can be associated with memory problems [46]. Further studies are needed to explore this premise.

In respect to alertness, scores were overall significantly higher/improved in the HOT compared to the CONTROL trial for the Active subgroup with no differences found between trials for the Less-Active subgroup. These improved scores were most likely related to the higher Tc values recorded for this trial/subgroup compared to CONTROL (p<0.05 main effect for trial), which most likely resulted in a warm-up effect. These results are supported by studies that reported improved alertness associated with increases in Tc that occurred as a result of changes in circadian rhythm [47–48]. Furthermore, while some studies have reported impaired alertness associated with hot environmental conditions [22–23], it would appear that the average peak Tc values recorded here during the HOT trials were not high enough to impair alertness. Notably, the higher alertness scores recorded in the HOT Active subgroup did not result in improved manual dexterity performance in these participants.

Heart-rate was also assessed in this study with results finding higher values (bpm) in the HOT compared to the CONTROL trial in both subgroups at most time points assessed. This outcome is most likely associated with the higher Tc values and significantly greater sweat loss found in the HOT trials, which together would have resulted in a higher heart-rate in order to maintain cardiac output due to reduced stroke volume [49]. Furthermore, as previously noted, thermal sensation was significantly higher in the HOT compared to the CONTROL trial in both subgroups. Of importance to surgical staff is that levels of frustration [24], as well as perceptions of distraction [25] have been reported to be greater in participants who felt hotter while performing surgical procedures as a result of a heated OR.

There are a number of limitations to this study. These include: the allocation of participants to an Active or Less-Active subgroup based on their surgery duties and subsequent observations rather than the use of tracking devices; the uneven and small size of the cohorts in these two subgroups; the use of a simulation (which might generate significantly less performance pressure than a real life operation); the grouping of male and female staff data; individual performance factor variability between trials; and the duration of the simulation being limited to 150 min. Furthermore, the inclusion of complex and simple cognitive testing and the assessment of surgeon performance represent areas that would be beneficial to include in later studies.

## Conclusion

Whilst working in a hot OR, staff felt hotter and experienced a significant loss in body-mass through sweat loss. Together this may have contributed to a slight impairment in manual dexterity, despite an improvement in alertness. It is possible that more significant differences may have been found between variables if a real life surgery that lasted longer than 150 min was employed. Results found here in respect to hydration/sweat loss suggest that staff working in a hot ambient surgery should attempt to be hydrated prior to surgery and should consider their fluid intake during surgery. Further studies are needed that focus on cooling and hydration of surgery staff during burn surgeries. As surgical staff represent the endurance athletes of the medical world it is important that their health status be optimal during surgeries.

## Supporting information

S1 File Manual dexterity, psychological and physiological raw data for Active versus Less Active and Hot versus CONTROL trials. (DOCX)
