# Supplementary material for: Effects of a hot ambient operating theatre on manual dexterity, psychological and physiological parameters in staff during a simulated burn surgery
Source: PLoS One. 2019 Oct 16;14(10):e0222923. doi: 10.1371/journal.pone.0222923 (PMC6795495; doi:10.1371/journal.pone.0222923)
Supplement: S1 File — (DOCX) [file pone.0222923.s001.docx]

**Table A in S1 File. CONTROL trial core temperature (^o^C) responses for Active (n=9) and Less Active (n=8) groups over entire trial (150 min).**

| **Time (min)** | **0** | **15** | **30** | **45** | **60** | **75** | **90** | **105** | **120** | **135** | **150** |
| --- | --- | --- | --- | --- | --- | --- | --- | --- | --- | --- | --- |
| **Active** |  |  |  |  |  |  |  |  |  |  |  |
| **1** | 36.39 | 36.61 | 36.73 | 36.94 | 37.05 | 37.28 | 37.40 | 37.62 | 37.71 | 37.75 | 37.77 |
| **2** | 37.43 | 37.63 | 37.76 | 37.81 | 37.85 | 37.88 | 37.85 | 37.85 | 37.77 | 37.81 | 37.72 |
| **3** | 37.42 | 37.54 | 37.62 | 37.46 | 37.52 | 37.49 | 37.55 | 37.42 | 37.42 | 37.43 | 37.48 |
| **4** | 37.69 | 37.43 | 37.61 | 37.66 | 37.66 | 37.56 | 37.49 | 37.49 | 37.49 | 37.34 | 37.40 |
| **5** | 37.36 | 37.14 | 37.20 | 37.26 | 37.30 | 37.28 | 37.27 | 37.24 | 37.30 | 37.26 | 37.22 |
| **6** | 36.94 | 36.82 | 36.99 | 36.89 | 36.90 | 37.04 | 37.10 | 37.31 | 37.37 | 37.41 | 37.41 |
| **7** | 37.05 | 37.25 | 37.35 | 37.25 | 37.25 | 37.40 | 37.05 | 37.45 | 37.05 | 37.45 | 37.15 |
| **8** | 37.74 | 37.82 | 37.70 | 37.77 | 37.79 | 37.94 | 37.97 | 37.88 | 37.94 | 37.91 | 37.83 |
| **9** | 36.99 | 37.04 | 37.05 | 37.05 | 37.19 | 37.15 | 37.14 | 37.21 | 37.27 | 37.23 | 37.14 |
| **Less Active** |  |  |  |  |  |  |  |  |  |  |  |
| **1** | 37.60 | 37.94 | 38.03 | 38.21 | 38.28 | 38.22 | 38.21 | 38.12 | 38.01 | 37.94 | 37.85 |
| **2** | 37.56 | 37.32 | 37.32 | 37.31 | 37.26 | 37.21 | 37.20 | 37.18 | 37.09 | 37.10 | 37.00 |
| **3** | 37.45 | 37.56 | 37.48 | 37.43 | 37.57 | 37.54 | 37.50 | 37.40 | 37.42 | 37.40 | 37.40 |
| **4** | 36.70 | 36.77 | 36.86 | 36.76 | 36.89 | 37.03 | 36.93 | 36.94 | 36.72 | 36.88 | 37.04 |
| **5** | 36.98 | 37.10 | 37.04 | 36.98 | 37.10 | 37.04 | 37.21 | 37.21 | 37.28 | 37.13 | 37.08 |
| **6** | 36.73 | 36.55 | 36.53 | 36.50 | 36.52 | 36.82 | 36.87 | 36.77 | 36.66 | 36.75 | 36.83 |
| **7** | 37.06 | 36.91 | 36.86 | 37.02 | 37.14 | 37.03 | 37.01 | 37.05 | 37.03 | 36.94 | 37.03 |
| **8** | 38.27 | 38.16 | 38.21 | 38.07 | 38.15 | 38.14 | 38.07 | 37.94 | 37.87 | 37.88 | 37.78 |

**Table B in S1 File. HOT trial core temperature (^o^C) responses for Active (n=9) and Less Active (n=8) groups over entire trial (150 min).**

| **Time (min)** | **0** | **15** | **30** | **45** | **60** | **75** | **90** | **105** | **120** | **135** | **150** |
| --- | --- | --- | --- | --- | --- | --- | --- | --- | --- | --- | --- |
| **Active** |  |  |  |  |  |  |  |  |  |  |  |
| **1** | 36.66 | 37.08 | 37.26 | 37.39 | 37.46 | 37.50 | 37.54 | 37.50 | 37.48 | 37.51 | 37.54 |
| **2** | 37.60 | 37.45 | 37.63 | 37.80 | 37.88 | 37.88 | 38.05 | 38.05 | 38.00 | 38.01 | 38.02 |
| **3** | 37.56 | 37.56 | 37.94 | 38.01 | 38.06 | 38.15 | 38.12 | 38.16 | 38.13 | 37.93 | 37.90 |
| **4** | 37.56 | 37.56 | 37.84 | 38.15 | 38.07 | 37.91 | 37.74 | 37.83 | 37.69 | 37.66 | 37.70 |
| **5** | 37.62 | 37.62 | 37.58 | 37.61 | 37.69 | 37.68 | 37.69 | 37.63 | 37.66 | 37.69 | 37.73 |
| **6** | 37.16 | 37.16 | 37.31 | 37.45 | 37.64 | 37.86 | 38.05 | 38.15 | 38.15 | 38.09 | 38.13 |
| **7** | 37.37 | 37.37 | 37.43 | 37.46 | 37.49 | 37.45 | 37.57 | 37.44 | 37.57 | 37.57 | 37.45 |
| **8** | 37.71 | 37.71 | 37.78 | 37.86 | 37.84 | 37.77 | 37.93 | 37.64 | 37.83 | 37.95 | 37.97 |
| **9** | 37.27 | 37.27 | 37.41 | 37.59 | 37.62 | 37.59 | 37.52 | 37.53 | 37.54 | 37.31 | 37.41 |
| **Less Active** |  |  |  |  |  |  |  |  |  |  |  |
| **1** | 37.79 | 38.01 | 38.10 | 38.11 | 38.06 | 38.05 | 38.09 | 38.09 | 38.03 | 38.01 | 38.06 |
| **2** | 37.39 | 37.27 | 37.30 | 37.31 | 37.34 | 37.33 | 37.37 | 37.38 | 37.37 | 37.37 | 37.39 |
| **3** | 37.67 | 37.55 | 37.50 | 37.58 | 37.65 | 37.67 | 37.70 | 37.64 | 37.64 | 37.67 | 37.72 |
| **4** | 36.54 | 36.54 | 36.82 | 36.96 | 37.14 | 37.09 | 37.29 | 37.43 | 37.46 | 37.41 | 37.37 |
| **5** | 37.15 | 37.15 | 37.29 | 37.37 | 37.39 | 37.34 | 37.34 | 37.40 | 37.50 | 37.46 | 37.48 |
| **6** | 37.00 | 37.00 | 37.10 | 37.25 | 37.38 | 37.46 | 37.40 | 37.34 | 37.29 | 37.29 | 37.34 |
| **7** | 37.12 | 37.12 | 37.11 | 37.22 | 37.30 | 37.40 | 37.35 | 37.35 | 37.31 | 37.31 | 37.35 |
| **8** | 37.56 | 37.56 | 37.51 | 37.45 | 37.76 | 37.86 | 38.06 | 38.17 | 38.12 | 38.06 | 38.17 |

**Table C in S1 File. CONTROL trial heart rate (bpm) responses for Active (n=9) and Less Active (n=8) groups over entire trial (150 min).**

| **Time (min)** | **0** | **15** | **30** | **45** | **60** | **75** | **90** | **105** | **120** | **135** | **150** |
| --- | --- | --- | --- | --- | --- | --- | --- | --- | --- | --- | --- |
| **Active** |  |  |  |  |  |  |  |  |  |  |  |
| **1** | 81 | 83 | 97 | 88 | 90 | 90 | 93 | 93 | 98 | 86 | 98 |
| **2** | 70 | 88 | 108 | 89 | 91 | 92 | 96 | 94 | 89 | 90 | 74 |
| **3** | 90 | 91 | 101 | 88 | 74 | 76 | 81 | 71 | 83 | 75 | 75 |
| **4** | 88 | 88 | 84 | 99 | 75 | 72 | 63 | 65 | 72 | 72 | 70 |
| **5** | 71 | 71 | 72 | 73 | 72 | 72 | 72 | 65 | 64 | 63 | 69 |
| **6** | 65 | 65 | 76 | 83 | 70 | 71 | 75 | 64 | 74 | 81 | 70 |
| **7** | 106 | 106 | 134 | 87 | 102 | 106 | 111 | 96 | 88 | 95 | 99 |
| **8** | 91 | 91 | 89 | 90 | 78 | 80 | 83 | 81 | 77 | 74 | 81 |
| **9** | 65 | 65 | 77 | 93 | 93 | 92 | 81 | 70 | 70 | 77 | 77 |
| **Less Active** |  |  |  |  |  |  |  |  |  |  |  |
| **1** | 114 | 118 | 127 | 109 | 106 | 96 | 101 | 102 | 95 | 83 | 93 |
| **2** | 66 | 79 | 61 | 63 | 58 | 55 | 66 | 53 | 56 | 61 | 69 |
| **3** | 84 | 85 | 82 | 81 | 78 | 71 | 80 | 83 | 78 | 72 | 74 |
| **4** | 69 | 69 | 81 | 54 | 53 | 80 | 59 | 61 | 69 | 68 | 63 |
| **5** | 68 | 68 | 91 | 71 | 72 | 76 | 63 | 67 | 63 | 68 | 72 |
| **6** | 69 | 69 | 94 | 71 | 71 | 67 | 62 | 70 | 65 | 65 | 65 |
| **7** | 75 | 75 | 72 | 79 | 74 | 74 | 78 | 72 | 74 | 73 | 73 |
| **8** | 118 | 118 | 102 | 115 | 100 | 108 | 105 | 97 | 92 | 102 | 112 |

**Table D in S1 File. HOT trial heart rate (bpm) responses for Active (n=9) and Less Active (n=8) groups over entire trial (150 min).**

| **Time (min)** | **0** | **15** | **30** | **45** | **60** | **75** | **90** | **105** | **120** | **135** | **150** |
| --- | --- | --- | --- | --- | --- | --- | --- | --- | --- | --- | --- |
| **Active** |  |  |  |  |  |  |  |  |  |  |  |
| **1** | 85 | 97 | 108 | 104 | 101 | 99 | 96 | 96 | 101 | 95 | 95 |
| **2** | 70 | 73 | 117 | 93 | 102 | 93 | 80 | 91 | 91 | 90 | 97 |
| **3** | 80 | 80 | 81 | 89 | 89 | 106 | 98 | 89 | 80 | 88 | 95 |
| **4** | 92 | 97 | 113 | 102 | 102 | 94 | 87 | 84 | 100 | 85 | 90 |
| **5** | 86 | 91 | 69 | 94 | 85 | 87 | 105 | 92 | 88 | 108 | 94 |
| **6** | 67 | 67 | 78 | 83 | 83 | 85 | 92 | 89 | 72 | 76 | 72 |
| **7** | 92 | 99 | 131 | 120 | 134 | 137 | 137 | 136 | 132 | 115 | 123 |
| **8** | 72 | 72 | 80 | 83 | 87 | 77 | 78 | 85 | 88 | 84 | 82 |
| **9** | 76 | 76 | 92 | 94 | 93 | 102 | 97 | 94 | 111 | 106 | 94 |
| **Less Active** |  |  |  |  |  |  |  |  |  |  |  |
| **1** | 114 | 122 | 129 | 127 | 117 | 116 | 108 | 116 | 118 | 116 | 112 |
| **2** | 75 | 75 | 74 | 64 | 70 | 63 | 60 | 74 | 72 | 68 | 71 |
| **3** | 79 | 64 | 70 | 68 | 71 | 70 | 63 | 63 | 71 | 70 | 68 |
| **4** | 53 | 53 | 69 | 67 | 67 | 61 | 60 | 65 | 68 | 58 | 66 |
| **5** | 95 | 99 | 117 | 95 | 98 | 93 | 98 | 104 | 98 | 100 | 100 |
| **6** | 68 | 68 | 87 | 88 | 80 | 86 | 78 | 82 | 84 | 78 | 84 |
| **7** | 85 | 91 | 86 | 91 | 99 | 92 | 95 | 79 | 92 | 93 | 87 |
| **8** | 90 | 100 | 107 | 120 | 111 | 107 | 100 | 94 | 98 | 111 | 102 |

**Table E in S1 File. Table** Mean ± SD core temperature and heart rate values at each 15-minute interval during the simulation for the Active (n=9) and the Less-Active (n=8) subgroups in the CONTROL and HOT trials.

| **CORE TEMPERATURE (ºC)** | | | | | | | | | | | |
| --- | --- | --- | --- | --- | --- | --- | --- | --- | --- | --- | --- |
|  | **0 min** | **15 min** | **30 min** | **45 min** | **60 min** | **75 min** | **90 min** | **105 min** | **120 min** | **135 min** | **150 min** |
| **CONTROL** | | | | | | | | | | | |
| ACTIVE | 37.22±0.40 | 37.25±0.37 | 37.33±0.34 | 37.34±0.33 | 37.39±0.31 | 37.43±0.30 | 37.42±0.31 | 37.50±0.23 | 37.48±0.26 | 37.51±0.24 | 37.46±0.25 |
| LESS ACTIVE | 37.29±0.50 | 37.29±0.53 | 37.29±0.55 | 37.29±0.56 | 37.36±0.57 | 37.38±0.50 | 37.38±0.48 | 37.33±0.44 | 37.26±0.46^a^ | 37.25±0.42^a^ | 37.25±0.36^a^ |
| **HOT** | | | | | | | | | | | |
| ACTIVE | 37.39±0.31 | 37.42±0.20 | 37.58±0.23 | 37.70±0.25 | 37.75±0.21 | 37.75±0.21 | 37.80±0.23 | 37.77±0.27 | 37.78±0.24 | 37.75±0.25 | 37.76±0.24 |
| LESS ACTIVE | 37.28±0.38 | 37.28±0.41 | 37.34±0.36^a^ | 37.41±0.32^a^ | 37.50±0.28^a^ | 37.53±0.29^a^ | 37.58±0.31^a^ | 37.60±0.32^a^ | 37.59±0.30^a^ | 37.57±0.29^a^ | 37.61±0.31^a^ |
| **HEART RATE (bpm)** | | | | | | | | | | | |
| **CONTROL** | | | | | | | | | | | |
| ACTIVE | 81 ± 13 | 83 ± 13 | 93 ± 19 | 88 ± 7 | 83 ± 11 | 83 ± 12 | 84 ± 14 | 78 ± 13 | 79 ± 10 | 79 ± 9 | 79 ± 11 |
| LESS ACTIVE | 83 ± 20 | 85 ± 20 | 89 ± 19 | 80 ± 20^a^ | 77 ± 17 | 78 ± 16 | 77 ± 17 | 76 ± 16 | 74 ± 13 | 74 ± 12 | 78 ± 16 |
| **HOT**^b^ | | | | | | | | | | | |
| ACTIVE | 80 ± 9 | 84 ± 12 | 97±20 | 96 ± 11 | 97 ± 15 | 98 ± 16 | 97 ± 17 | 95 ± 15 | 96 ± 17 | 94 ± 12 | 94 ± 13 |
| LESS ACTIVE | 82 ± 17 | 84 ± 21 | 92±21 | 90 ± 22 | 89 ± 18 | 86 ± 19^a^ | 83 ± 19^a^ | 85 ± 18^a^ | 88 ± 16 | 87 ± 20 | 86 ± 16^a^ |
|  |  |  |  |  |  |  |  |  |  |  |  |

^a^ indicates moderate to large effect size between trials in the ACTIVE and LESS-ACTIVE subgroups (*d*=0.55 to 1.02)

^b^ indicates significant interaction effect between ACTIVE and LESS-ACTIVE subgroups (p<0.05)

**Table F in S1 File. Urinary Specific Gravity prior to HOT and CONTROL trials in Active (n=9) and Less Active (n=8) groups.**

|  | **CONTROL** | **HOT** |
| --- | --- | --- |
| **Active** |  |  |
| **1** | 1.020 | 1.020 |
| **2** | 1.015 | 1.015 |
| **3** | 1.010 | 1.005 |
| **4** | 1.020 | 1.010 |
| **5** | 1.005 | 1.015 |
| **6** | 1.015 | 1.015 |
| **7** | 1.020 | 1.010 |
| **8** | 1.025 | 1.005 |
| **9** | 1.005 | 1.010 |
| **Less Active** |  |  |
| **1** | 1.010 | 1.010 |
| **2** | 1.005 | 1.010 |
| **3** | 1.005 | 1.005 |
| **4** | 1.020 | 1.025 |
| **5** | 1.005 | 1.020 |
| **6** | 1.020 | 1.005 |
| **7** | 1.020 | 1.020 |
| **8** | 1.010 | 1.020 |

**Table G in S1 File. Body-mass changes (pre- and post-mass; kg) over HOT and CONTROL trials in Active (n=9) and Less Active (n=8) groups.**

|  | **CONTROL** | | **HOT** | |
| --- | --- | --- | --- | --- |
|  | Pre Mass | Post Mass | Pre Mass | Post Mass |
| **Active** |  |  |  |  |
| **1** | 61.0 | 60.6 | 61.7 | 61.1 |
| **2** | 57.9 | 57.8 | 57.6 | 56.9 |
| **3** | 79.7 | 79.5 | 81.4 | 80.8 |
| **4** | 54.0 | 53.8 | 55.5 | 55.3 |
| **5** | 54.5 | 54.3 | 54.9 | 54.5 |
| **6** | 69.7 | 69.6 | 70.0 | 69.6 |
| **7** | 64.1 | 64.0 | 63.0 | 62.4 |
| **8** | 100.8 | 100.2 | 103.1 | 101.8 |
| **9** | 71.6 | 71.3 | 69.3 | 68.5 |
| **Less Active** |  |  |  |  |
| **1** | 89.0 | 88.8 | 90.5 | 89.8 |
| **2** | 84.8 | 84.5 | 86.7 | 85.9 |
| **3** | 76.3 | 76.0 | 77.7 | 77.2 |
| **4** | 69.6 | 69.6 | 70.1 | 68.6 |
| **5** | 89.0 | 88.9 | 88.7 | 87.7 |
| **6** | 59.5 | 59.4 | 59.6 | 59.3 |
| **7** | 75.2 | 75.1 | 75.1 | 74.8 |
| **8** | 127.1 | 126.5 | 126.2 | 125.2 |

**Table H in S1 File. CONTROL trial ratings of thermal sensation for Active (n=9) and Less Active (n=8) groups over entire trial (150 min).**

| **Time (min)** | **0** | **30** | **60** | **90** | **120** | **150** |
| --- | --- | --- | --- | --- | --- | --- |
| **Active** |  |  |  |  |  |  |
| **1** | 2.0 | 4.5 | 5.5 | 6.0 | 6.0 | 6.5 |
| **2** | 3.0 | 5.0 | 6.0 | 6.0 | 6.0 | 6.0 |
| **3** | 3.5 | 3.5 | 4.0 | 4.5 | 4.0 | 4.0 |
| **4** | 4.0 | 4.5 | 4.0 | 3.5 | 4.0 | 4.0 |
| **5** | 4.0 | 5.0 | 5.0 | 4.0 | 4.0 | 4.0 |
| **6** | 3.0 | 2.5 | 3.5 | 4.0 | 4.0 | 3.5 |
| **7** | 3.0 | 4.0 | 4.0 | 4.5 | 5.0 | 3.5 |
| **8** | 3.0 | 3.0 | 3.5 | 4.5 | 4.0 | 3.5 |
| **9** | 3.5 | 3.5 | 3.5 | 4.0 | 4.0 | 3.5 |
| **Less Active** |  |  |  |  |  |  |
| **1** | 5.0 | 6.0 | 6.5 | 6.0 | 5.0 | 5.0 |
| **2** | 4.5 | 4.0 | 4.0 | 3.5 | 4.0 | 4.0 |
| **3** | 3.5 | 5.0 | 5.0 | 5.0 | 5.5 | 5.5 |
| **4** | 3.5 | 3.5 | 3.0 | 3.5 | 3.5 | 3.0 |
| **5** | 4.5 | 4.5 | 5.0 | 4.5 | 4.0 | 4.0 |
| **6** | 4.0 | 4.0 | 4.0 | 4.0 | 4.0 | 4.0 |
| **7** | 4.0 | 4.0 | 4.0 | 3.5 | 4.0 | 4.0 |
| **8** | 5.0 | 6.0 | 6.5 | 6.0 | 5.0 | 6.5 |

**Table I in S1 File. HOT trial ratings of thermal sensation for Active (n=9) and Less Active (n=8) groups over entire trial (150 min).**

| **Time (min)** | **0** | **30** | **60** | **90** | **120** | **150** |
| --- | --- | --- | --- | --- | --- | --- |
| **Active** |  |  |  |  |  |  |
| **1** | 6.0 | 6.5 | 6.5 | 7.0 | 6.5 | 6.0 |
| **2** | 6.0 | 7.0 | 7.0 | 7.0 | 7.0 | 7.0 |
| **3** | 4.5 | 5.5 | 5.5 | 5.5 | 6.0 | 6.0 |
| **4** | 5.0 | 6.0 | 6.0 | 6.0 | 6.0 | 7.0 |
| **5** | 5.0 | 5.5 | 5.5 | 5.0 | 5.5 | 5.0 |
| **6** | 5.0 | 5.0 | 5.0 | 6.0 | 6.5 | 4.5 |
| **7** | 5.5 | 5.5 | 5.5 | 6.5 | 6.0 | 6.0 |
| **8** | 6.5 | 6.5 | 6.5 | 6.0 | 6.5 | 6.0 |
| **9** | 4.0 | 5.0 | 6.0 | 6.0 | 5.5 | 5.0 |
| **Less Active** |  |  |  |  |  |  |
| **1** | 6.5 | 7.0 | 7.5 | 7.5 | 7.5 | 7.5 |
| **2** | 5.0 | 5.0 | 5.5 | 5.5 | 5.5 | 6.0 |
| **3** | 5.5 | 5.5 | 5.5 | 6.0 | 6.0 | 6.0 |
| **4** | 5.0 | 5.5 | 6.0 | 6.0 | 6.0 | 5.5 |
| **5** | 6.5 | 6.5 | 6.5 | 7.0 | 6.0 | 6.0 |
| **6** | 7.0 | 7.0 | 7.5 | 7.5 | 7.5 | 7.5 |
| **7** | 5.0 | 5.0 | 5.5 | 5.5 | 5.0 | 4.5 |
| **8** | 6.0 | 7.0 | 7.0 | 7.0 | 7.5 | 7.5 |

**Table J in S1 File. CONTROL trial alertness responses/scores Stanford Sleepiness Scale (scale 1-7) for Active (n=9) and Less Active (n=8) groups over entire trial (150 min).**

| **Time (min)** | **0** | **30** | **60** | **90** | **120** | **150** |
| --- | --- | --- | --- | --- | --- | --- |
| **Active** |  |  |  |  |  |  |
| **1** | 6 | 7 | 7 | 7 | 7 | 7 |
| **2** | 7 | 6 | 6 | 5 | 5 | 4 |
| **3** | 4 | 5 | 5 | 4 | 3 | 5 |
| **4** | 5 | 6 | 6 | 6 | 6 | 7 |
| **5** | 6 | 6 | 6 | 6 | 5 | 5 |
| **6** | 3 | 5 | 5 | 5 | 5 | 5 |
| **7** | 5 | 6 | 6 | 6 | 5 | 5 |
| **8** | 5 | 6 | 6 | 7 | 7 | 7 |
| **9** | 3 | 3 | 5 | 5 | 5 | 5 |
| **Less Active** |  |  |  |  |  |  |
| **1** | 7 | 7 | 6 | 5 | 5 | 5 |
| **2** | 6 | 6 | 6 | 6 | 6 | 6 |
| **3** | 5 | 6 | 6 | 5 | 5 | 5 |
| **4** | 5 | 6 | 5 | 5 | 6 | 6 |
| **5** | 5 | 6 | 5 | 5 | 5 | 5 |
| **6** | 6 | 6 | 6 | 6 | 6 | 6 |
| **7** | 2 | 4 | 5 | 5 | 5 | 6 |
| **8** | 6 | 6 | 6 | 6 | 6 | 6 |

**Table K in S1 File. HOT trial alertness responses/scores Stanford Sleepiness Scale (scale 1-7) for Active (n=9) and Less Active (n=8) groups over entire trial (150 min).**

| **Time (min)** | **0** | **30** | **60** | **90** | **120** | **150** |
| --- | --- | --- | --- | --- | --- | --- |
| **Active** |  |  |  |  |  |  |
| **1** | 5 | 6 | 6 | 7 | 7 | 7 |
| **2** | 6 | 6 | 6 | 6 | 3 | 3 |
| **3** | 6 | 6 | 7 | 7 | 7 | 7 |
| **4** | 5 | 6 | 7 | 7 | 6 | 6 |
| **5** | 6 | 7 | 7 | 6 | 6 | 5 |
| **6** | 6 | 6 | 7 | 7 | 7 | 7 |
| **7** | 5 | 7 | 7 | 7 | 7 | 7 |
| **8** | 6 | 6 | 6 | 7 | 7 | 7 |
| **9** | 5 | 6 | 6 | 6 | 6 | 6 |
| **Less Active** |  |  |  |  |  |  |
| **1** | 7 | 7 | 7 | 7 | 6 | 6 |
| **2** | 6 | 6 | 5 | 5 | 5 | 4 |
| **3** | 5 | 6 | 6 | 6 | 7 | 6 |
| **4** | 6 | 6 | 6 | 6 | 6 | 6 |
| **5** | 5 | 5 | 5 | 5 | 5 | 5 |
| **6** | 5 | 7 | 6 | 6 | 6 | 6 |
| **7** | 4 | 4 | 4 | 4 | 4 | 3 |
| **8** | 5 | 6 | 6 | 4 | 6 | 4 |

**Table L in S1 File. CONTROL trial Purdue pegboard scores (dominant hand) for Active (n=9) and Less Active (n=8) groups over entire trial (150 min).**

| **Time (min)** | **0** | **30** | **60** | **90** | **120** | **150** |
| --- | --- | --- | --- | --- | --- | --- |
| **Active** |  |  |  |  |  |  |
| **1** | 15 | 18 | 20 | 20 | 20 | 20 |
| **2** | 18 | 17 | 18 | 18 | 19 | 19 |
| **3** | 16 | 14 | 15 | 15 | 16 | 18 |
| **4** | 21 | 19 | 22 | 20 | 21 | 21 |
| **5** | 18 | 17 | 16 | 16 | 17 | 19 |
| **6** | 17 | 15 | 16 | 16 | 14 | 15 |
| **7** | 15 | 15 | 15 | 17 | 15 | 17 |
| **8** | 14 | 16 | 16 | 18 | 16 | 16 |
| **9** | 18 | 18 | 18 | 18 | 19 | 18 |
| **Less Active** |  |  |  |  |  |  |
| **1** | 13 | 17 | 16 | 16 | 16 | 15 |
| **2** | 14 | 14 | 16 | 17 | 15 | 14 |
| **3** | 14 | 16 | 16 | 16 | 16 | 17 |
| **4** | 18 | 17 | 18 | 19 | 19 | 20 |
| **5** | 17 | 19 | 19 | 18 | 18 | 17 |
| **6** | 18 | 18 | 18 | 16 | 17 | 18 |
| **7** | 19 | 17 | 18 | 17 | 17 | 14 |
| **8** | 16 | 17 | 18 | 18 | 17 | 18 |

**Table M in S1 File. HOT trial Purdue pegboard scores (dominant hand) for Active (n=9) and Less Active (n=8) groups over entire trial (150 min).**

| **Time (min)** | **0** | **30** | **60** | **90** | **120** | **150** |
| --- | --- | --- | --- | --- | --- | --- |
| **Active** |  |  |  |  |  |  |
| **1** | 18 | 19 | 20 | 21 | 22 | 21 |
| **2** | 18 | 19 | 20 | 19 | 20 | 19 |
| **3** | 18 | 14 | 16 | 15 | 15 | 17 |
| **4** | 18 | 18 | 20 | 20 | 18 | 22 |
| **5** | 16 | 18 | 17 | 18 | 18 | 18 |
| **6** | 11 | 15 | 15 | 17 | 15 | 16 |
| **7** | 14 | 13 | 13 | 18 | 17 | 18 |
| **8** | 14 | 15 | 16 | 17 | 16 | 14 |
| **9** | 14 | 16 | 17 | 19 | 20 | 19 |
| **Less Active** |  |  |  |  |  |  |
| **1** | 16 | 18 | 15 | 14 | 16 | 15 |
| **2** | 16 | 17 | 16 | 15 | 17 | 15 |
| **3** | 15 | 18 | 18 | 18 | 18 | 18 |
| **4** | 17 | 14 | 16 | 16 | 17 | 18 |
| **5** | 13 | 17 | 17 | 18 | 15 | 16 |
| **6** | 15 | 17 | 17 | 18 | 17 | 18 |
| **7** | 15 | 17 | 17 | 17 | 17 | 17 |
| **8** | 18 | 18 | 16 | 20 | 18 | 19 |

**Table N in S1 File. CONTROL trial Purdue pegboard scores (non-dominant hand) for Active (n=9) and Less Active (n=8) groups over entire trial (150 min).**

| **Time (min)** | **0** | **30** | **60** | **90** | **120** | **150** |
| --- | --- | --- | --- | --- | --- | --- |
| **Active** |  |  |  |  |  |  |
| **1** | 12 | 17 | 16 | 19 | 17 | 16 |
| **2** | 19 | 18 | 18 | 17 | 17 | 20 |
| **3** | 15 | 15 | 14 | 13 | 15 | 15 |
| **4** | 18 | 19 | 18 | 19 | 19 | 20 |
| **5** | 15 | 16 | 17 | 16 | 18 | 16 |
| **6** | 14 | 14 | 14 | 15 | 15 | 16 |
| **7** | 14 | 14 | 16 | 17 | 17 | 15 |
| **8** | 14 | 18 | 19 | 18 | 19 | 16 |
| **9** | 17 | 17 | 18 | 19 | 17 | 17 |
| **Less Active** |  |  |  |  |  |  |
| **1** | 15 | 15 | 16 | 15 | 13 | 14 |
| **2** | 13 | 13 | 13 | 12 | 14 | 15 |
| **3** | 14 | 14 | 14 | 16 | 16 | 12 |
| **4** | 13 | 15 | 13 | 14 | 13 | 15 |
| **5** | 18 | 17 | 17 | 18 | 17 | 20 |
| **6** | 15 | 17 | 16 | 16 | 16 | 17 |
| **7** | 15 | 15 | 16 | 18 | 17 | 18 |
| **8** | 15 | 16 | 15 | 17 | 17 | 16 |

**Table O in S1 File. HOT trial Purdue pegboard scores (non-dominant hand) for Active (n=9) and Less Active (n=8) groups over entire trial (150 min).**

| **Time (min)** | **0** | **30** | **60** | **90** | **120** | **150** |
| --- | --- | --- | --- | --- | --- | --- |
| **Active** |  |  |  |  |  |  |
| **1** | 16 | 20 | 20 | 16 | 20 | 19 |
| **2** | 18 | 18 | 19 | 18 | 21 | 19 |
| **3** | 15 | 14 | 14 | 15 | 15 | 17 |
| **4** | 13 | 16 | 16 | 18 | 17 | 19 |
| **5** | 17 | 17 | 17 | 17 | 15 | 16 |
| **6** | 13 | 14 | 15 | 13 | 15 | 15 |
| **7** | 14 | 13 | 14 | 15 | 16 | 17 |
| **8** | 18 | 18 | 18 | 19 | 18 | 18 |
| **9** | 15 | 16 | 16 | 17 | 17 | 17 |
| **Less Active** |  |  |  |  |  |  |
| **1** | 15 | 14 | 15 | 13 | 15 | 14 |
| **2** | 13 | 15 | 15 | 15 | 15 | 16 |
| **3** | 15 | 15 | 17 | 17 | 16 | 16 |
| **4** | 13 | 12 | 11 | 13 | 13 | 14 |
| **5** | 14 | 15 | 18 | 19 | 17 | 17 |
| **6** | 17 | 17 | 17 | 16 | 16 | 17 |
| **7** | 16 | 13 | 16 | 13 | 15 | 14 |
| **8** | 15 | 14 | 15 | 16 | 16 | 16 |
